# Genome-Wide Association Study Reveals Quantitative Trait Loci and Candidate Genes Associated with High Interferon-gamma Production in Holstein Cattle Naturally Infected with *Mycobacterium Bovis*

**DOI:** 10.3390/ijms25116165

**Published:** 2024-06-03

**Authors:** Gerard Badia-Bringué, María Canive, Patricia Vázquez, Joseba M. Garrido, Almudena Fernández, Ramón A. Juste, José Antonio Jiménez, Oscar González-Recio, Marta Alonso-Hearn

**Affiliations:** 1 Department of Animal Health, NEIKER-Basque Institute for Agricultural Research and Development, Basque Research and Technology Alliance (BRTA), 48160 Derio, Bizkaia, Spain; 2 Departamento de Mejora Genética Animal, Instituto Nacional de Investigación y Tecnología Agraria y Alimentaria (INIA), 28040 Madrid, Spain; 3 CONAFE-Spanish Confederation of Holstein Cattle, 28340 Madrid, Spain

**Keywords:** *Mycobacterium bovis*, interferon-gamma, polymorphisms, breeding

## Abstract

*Mycobacterium bovis* (*Mb*) is the causative agent of bovine tuberculosis (bTb). Genetic selection aiming to identify less susceptible animals has been proposed as a complementary measure in ongoing programs toward controlling *Mb* infection. However, individual animal phenotypes for bTb based on interferon-gamma (IFNɣ) and its use in bovine selective breeding programs have not been explored. In the current study, IFNɣ production was measured using a specific IFNɣ ELISA kit in bovine purified protein derivative (bPPD)-stimulated blood samples collected from Holstein cattle. DNA isolated from the peripheral blood samples collected from the animals included in the study was genotyped with the EuroG Medium Density bead Chip, and the genotypes were imputed to whole-genome sequences. A genome-wide association analysis (GWAS) revealed that the IFNɣ in response to bPPD was associated with a specific genetic profile (heritability = 0.23) and allowed the identification of 163 SNPs, 72 quantitative trait loci (QTLs), 197 candidate genes, and 8 microRNAs (miRNAs) associated with this phenotype. No negative correlations between this phenotype and other phenotypes and traits included in the Spanish breeding program were observed. Taken together, our results define a heritable and distinct immunogenetic profile associated with strong production of IFNɣ in response to *Mb*.

## 1. Introduction

Animal tuberculosis is a chronic infection caused by members of the *Mycobacterium tuberculosis* complex (MTC). *Mycobacterium bovis* (*Mb*), an intracellular, Gram-positive pathogen, is the key pathogen responsible for bovine tuberculosis (bTb). This zoonotic disease causes important economic losses worldwide, is considered a threat to public health, and has implications for the international trade of animals [1,2]. 

*Mb* resides within phagosomes in infected host macrophages [3]. Soon after being infected, the macrophages produce interleukin 12 (IL12), which activates natural killer (NK) cells and T lymphocytes to produce interferon-gamma (IFNɣ), leading to the activation of macrophages, enhanced release of nitric oxide, and restricted bacterial multiplication [4]. However, *Mb* has evolved ways to evade the host defense and to replicate within infected macrophages by inhibiting phagosome–lysosome fusion. *Mb* suppresses the antimicrobial immune response in macrophages, facilitating intracellular survival and immune evasion through autophagy inhibition and macrophage M2 polarization [5]. In advanced stages of infection, IFNγ is involved in the pathogenesis of several immunological disorders caused by unrestrained inflammatory responses. In a vaccination–challenge study, Vordermeier et al. found that following a challenge with *Mb*, the IFNγ response to the early secreted antigenic target 6 kDa antigen (ESAT-6) correlated positively with lesion scores in infected animals [6]. On the other hand, previous studies have shown that IFNγ production might be regulated post-transcriptionally by micro-RNAs (miRNAs). For instance, the downregulation of miRNA-29 has been shown to upregulate IFNɣ-mediated innate and adaptative responses to *Mb* infection [7]. Transgenic mice in which the miRNA-29 was knocked down initiated a more potent IFNγ production in activated NK and T cells after *Mb* infection. 

Monitoring, control, and eradication programs for bTb have been established in multiple countries. Vaccination is not used as a preventive measure in animals because of the potential interference with bTb surveillance and diagnostic tests. In Spain, all regions are subjected to a national eradication program based on animal reaction to the single intradermal tuberculin test (SITT), the slaughter of reactor animals, and post-mortem confirmation of positive animals with histopathological lesions compatible with bTb. In some countries, including Spain, IFNγ release assays (IGRAs) are being used for diagnosis in combination with the single intradermal comparative cervical test (SICCT) [8]. The sensitivity of the SITT was 63.7% (95% CI, 54.54–72.00), while the sensitivity of the IFNγ assays ranged between 60.2% and 92% [9]. Results from experimental and natural infections of cattle with *Mb* indicated that the IGRA can detect a cell-mediated immune response (CMI) to infection as early as two weeks post-infection, earlier than the SICCT [10,11,12,13]. In general, however, bTb diagnostic tests are unable to distinguish the infection from the disease [14]. A study by Bernitz et al. [15] observed that levels of IFNγ in unstimulated whole blood were elevated in infected buffaloes with observable pathological changes consistent with bTb in comparison with uninfected controls. Furthermore, increased IFNγ significantly correlated with increasing severity of pathological changes in the infected buffaloes, consistent with observations of associations between antigen-stimulated IFNγ and bTb pathology in cattle and badgers, demonstrating the potential of this cytokine to be used as an indicator of bTb [16,17]. 

The incidence of new cases of bTb in parts of Spain suggests that existing control strategies are insufficient to eradicate the disease and that additional control measures that can complement current strategies need to be explored [18,19,20]. Currently, there are no vaccines commercially available that allow differentiation between naturally infected and vaccinated individuals. Genetic selection aiming to identify and select more resistant or less susceptible animals has been proposed as an additional measure in ongoing programs toward controlling bTb [21]. The effects of this strategy are cumulative and permanent, and they are transferred to subsequent generations and might result in disease eradication. 

BTb is a multifactorial disease that is the result of the interaction of genetic, environmental, and microbial factors. Previous studies quantified the genetic variation for bTb in different cattle populations and countries using a variety of trait definitions and reported heritability estimates that ranged between 0.06 and 0.18 [22,23,24,25]. More recent studies using genome-wide DNA arrays reported higher heritability estimates of 0.21–0.27 [26,27,28,29,30]. These studies demonstrated that host genetics plays an important role in the susceptibility/resistance to *Mb* infection and, therefore, a breeding strategy focused on increasing resistance or reducing susceptibility to *Mb* infection is feasible and currently used by farmers in some countries such as the UK and Ireland [21,25]. Different genetic models were investigated, and the single-step best linear unbiased prediction (BLUP) model resulted in the most accurate estimates of animal genetic merit for bTb resistance [31]. 

Defining the adequate phenotype is the main challenge in identifying the genetic profile of resistance or susceptibility against an infection. In the case of bTb, most previous studies combined SITT tests and postmortem data, such as records of bTb lesions and *Mb* bacteriological culture, to define the health status of each animal. Recently, reductionist approaches have been used to investigate a host biological subsystem, such as a key cellular function, whose performance is the phenotypical criterion for the classification of the population [32]. By performing a genome-wide association analysis (GWAS), our research group identified a total of 71 single-nucleotide polymorphisms (SNPs) associated with significant production of IFNγ in avian tuberculin-stimulated blood samples from *Mycobacterium avium* subsp. *paratuberculosis* (MAP)-infected cattle using whole-genome sequence (WGS) data [33]. In the current study, we hypothesize that animals able to induce a strong IFNɣ in response to *Mb* infection might also have specific host genetics. This study aimed to identify SNPs, quantitative trait loci (QTL), and candidate genes associated with susceptibility or resistance to *Mb* infection using IFNɣ production in response to bovine tuberculin as an indicator. The identified QTLs were compared with reported and annotated QTLs associated with health, body conformation, milk production, meat and carcass, reproduction, and length of productive life. In addition, the identified candidate genes were compared with bovine and human candidate genes previously associated with bovine and human tuberculosis, respectively. Undesirable genetic linkages between IFNɣ production and other traits included in the Spanish Holstein cattle evaluations were assessed. For this purpose, genomic estimated breeding values (gEBVs) for IFNɣ production were estimated in a larger independent population (N = 1739), and the correlations with 65 traits and phenotypes included in the Spanish evaluations of Holstein cattle were analyzed. 

## 2. Materials and Methods

### 2.1. Animals and Disease Status

The animals included in this study belonged to a reference population of 986 culled Holstein cattle that were slaughtered from March 2007 to May 2010. To ensure that the animals had a mature immune system, the cows included in the reference population were older than 2 years. Sampling was systematically performed once a week at the slaughterhouse. In each visit, the first 2 to 10 animals satisfying the breed and age requirements were sampled (on average 5 animals/sampling). The cows were slaughtered in the Bilbao and Donostia municipal slaughterhouses (Basque Country, Spain) under the pertinent Basque (Basque Government Decree 454/1994), Spanish (Spanish Government Law 32/2007 and Royal decree 731/2007), and European (Council Regulation No. 1099/2009) legislation on animal welfare. The cows were not submitted to any in vivo experimentation; therefore, no specific ethical authorization was needed. 

### 2.2. Interferon-Gamma Release Assay (IGRA)

For the IGRA, blood stimulation must be performed within the first eight hours after blood collection. Since one of the abattoirs was far from our research institute, IGRA could be performed in blood samples from only 343 of the 986 culled cows. Blood stimulation was performed as previously described [33,34]. Briefly, four 1.4 mL aliquots of lithium heparinized whole blood samples from each animal were added to four wells of a 24-well plate (Becton Dickinson, Franklin Lakes, NJ, USA). The blood samples were then stimulated with 100 µL of phosphate-buffered saline (PBS), 100 µL of avian purified protein derivative (aPPD) (0.3 µg/µL) (CZ Vaccines^®^ SA, Porriño, Spain), 100 µL of bovine purified protein derivative (bPPD) (0.3 µg/µL) (CZ Vaccines^®^ SA, Porriño, Spain), and 100 µL of lectin (1 μg/mL) as a positive control. BPPD is derived from *Mb*, strain AN-5. After incubating for 16 to 24 h at 37 °C in a 5% CO_2_ incubator, the plasma was separated by centrifugation at 500× *g* for 10 min at room temperature (RT) and then was frozen at −20 °C until testing. Subsequently, IFNɣ levels were measured in triplicate from the plasma samples using a specific IFNɣ ELISA test according to the manufacturer’s instructions (Bovigam^TM^, Prionics, Schlieren, Switzerland). IFNɣ levels were expressed as the OD of the aPPD or bPPD-stimulated plasmas minus the OD of the PBS-stimulated samples. In the current study, 117 cows from the 343 animals with an IGRA record were selected because they had an OD (bPPD-PBS) higher than the OD (aPPD-PBS) and the OD (bPPD) was higher than the OD (PBS) as well. This selection avoids false positive reactions with MAP infection and negative OD values, respectively. According to the interpretation criteria of the kit, 76 of the 117 cows included in the study were bTb-positive because the OD of the bPPD-PBS was >0.05 and the OD (bPPD-PBS) was higher than the OD (aPPD-PBS). 

### 2.3. Genotyping and Imputation to Whole-Genome Sequence (WGS)

Peripheral blood (PB) samples were collected into 10 mL Vacutainer EDTA tubes (Becton Dickinson, Franklin Lakes, NJ, USA) at the time of slaughter. Total DNA was extracted from the PB samples using the QIAmp DNA Blood Mini Kit according to the manufacturer’s instructions (Qiagen, Hilden, Germany). Purified DNA was then quantified by spectrophotometry and genotyped using the EuroG Medium-Density Bead Chip (Illumina) at the molecular genetic laboratory service of the Spanish Federation of Holstein Cattle (CONAFE) using the *InfiniumTM iScan* software for allele assignation (Illumina, San Diego, CA, USA). The individual genotypes were imputed to WGS as previously described [35]. Briefly, genotypes were phased using *Eagle 2.4* [36] and imputed with *minimac4* [37] to the Bovine High-Density Bead Chip (581,712 SNPs) using a reference panel of 1278 Holstein bulls from Run7.0 of the 1000 Bull Genomes project. Imputation to the WGS level (ARS-UCD1.2) was then undertaken using the same phasing and imputation procedure and a reference population of 2333 *Bos taurus* from Run7.0 of the 1000 Bull Genomes project [38]. Finally, the following filters were applied: call rate > 0.80, minimum allele frequency (MAF) > 0.01, and imputation score (r^2^) > 0.7. The final number of SNPs per animal was 11,122,500.

### 2.4. GWAS Analysis, Variance Components, and h^2^ Estimation

The IFNɣ levels in response to the bPPD, OD (bPPD-PBS), was the quantitative phenotype in the GWAS analysis. The variance components and h^2^ explained by all the SNPs were calculated using the genome-wide complex trait analysis (*GCTA*) software 1.93.2 [39], according to the following formula:

$$h^{2}=\frac{\sigma_{G}^{2}\;}{\sigma_{G}^{2}+\sigma_{e}^{2}}$$

where $\sigma_{G}^{2}$ represents the variance explained by all the SNPs and $\sigma_{e}^{2}$ the residual variance. These WGS data and the OD of these bPPD-PBS data were analyzed using the mixed linear model association analysis of the *GCTA* 1.93.2 software, expressed as *y = a + bx + g + e*. In the model, *y* is the phenotype, *a* is the mean term, *b is* the allele effect, *x is* the genotype of the SNP coded as 0, 1, or 2 depending on the copies of the minor allele, *g* is the polygenic effect as a random effect (assumed to be distributed as N~(0,$\;\sigma_{e}^{2}$), and *e* is the residual effect (also assumed to be distributed as N~(0,$\;\sigma_{e}^{2}$)). Age was included as a covariate in the analysis. To account for multiple testing, a 5% genome-wide false discovery rate (FDR) was used. A threshold of *p*-value ≤ 5 × 10^−7^ was used as suggested by the Wellcome Trust Case Control Consortium [40]. The inflation factor (*λ*) and quantile–quantile plots were used to compare the observed distributions of the −log (*p*-values) to the expected distribution under the no-association model. *λ* values close to 1 suggest appropriate adjustment for the potential substructure, and *λ* > 1.2 suggests population stratification. The SNP effects (*b*-values) were also calculated using the *GCTA* 1.93.2 software. If the sign of the *b*-value is positive, it implies that there is a positive relationship between the variables SNP and IFNɣ levels.

### 2.5. GWAS Data Post-Processing

SNPs identified in the GWAS analysis were filtered based on the *p*-values and clumping. Briefly, clumping is a process that first selects the most significant SNP and clumps (removes) other SNPs in a particular window that are in linkage disequilibrium with the selected SNP. The clumping was performed using the software *PLINK1.9* [41] with a window of 500 Kbp and a linkage disequilibrium-based correlation index (r^2^) of 0.9. 

### 2.6. SNPs, Quantitative Trait Loci (QTLs), QTLs Enrichment Analysis, Candidate Genes Identification, and Protein-to-Protein Interaction Networks

After clumping, the localization of the significant SNPs (FDR ≤ 0.05, *p*-value ≤ 5 × 10^−7^), QTLs, and candidate genes was performed using the ARS-UCD1.3 reference genome as previously described [33]. The genomic localization of the identified SNPs in the ARS-UCD1.3 reference genome was determined using the *Ensembl Variant Effect Predictor* (VEP) [42].

The R package *Genomic functional Annotation in Livestock for positional candidate Loci* (GALLO) [43] was used to identify in the cattle QTL database release 52 [44] annotated QTLs within an interval of 500 Kbp of the identified SNPs. Overlapping QTLs were merged to create a single QTL. To determine which of the annotated QTLs were overrepresented, a QTL enrichment analysis was performed using GALLO. 

Candidate genes within the QTLs were identified using Ensembl [42]. Candidate genes were compared with bovine and human candidate genes that were previously associated with bovine and human tuberculosis [45]. The function of the candidate genes was searched in *GeneCards* [46] by searching their gene symbol. To investigate the potential innate immune function of the identified candidate genes further, we searched the Innate DB database [47]. Protein-to-protein interaction networks were analyzed using *String* v2.0., setting the minimum required interaction score to 0.7.

### 2.7. Genomic Estimated Breeding Values (gEBVs) for IFNγ Production in Response to Bovine Tuberculin and Correlations with Other Bovine Phenotypes and Traits

gEBVs for IFNγ production were calculated for each animal in the study population based on the effect of each SNP with evidence of association with the IFNγ production (P_FDR_ ≤ 0.05) using the genomic best linear unbiased prediction (gBLUP) model of *GCTA* v1.93.2. [39,48]. Subsequently, gEBVs for IFNγ production were predicted in a larger population of 1739 Friesian cattle. Correlations between the gEBVs for IFNγ levels and 65 phenotypes and traits included in the evaluations of Spanish Holstein cattle were calculated in the larger population (N = 1739) with the Spearman’s rank correlation (ρ) implemented in R v4.1.2.

## 3. Results

### 3.1. Assessment of IFNɣ Production in Response to Bovine Tuberculin Stimulation

Only 117 of the 343 cows with an IGRA record were included in the study because they had an OD (bPPD-PBS) higher than the OD (aPPD-PBS), and the OD (bPPD) was higher than the OD (PBS). IFNɣ levels after blood stimulation with bPPD (OD (bPPD-PBS)) took values between 0.01 and 1.99, with most of the animals showing OD values between 0.01 and 0.5. The mean OD (bPPD-PBS) of the population was 0.17. 

**Figure 1:**
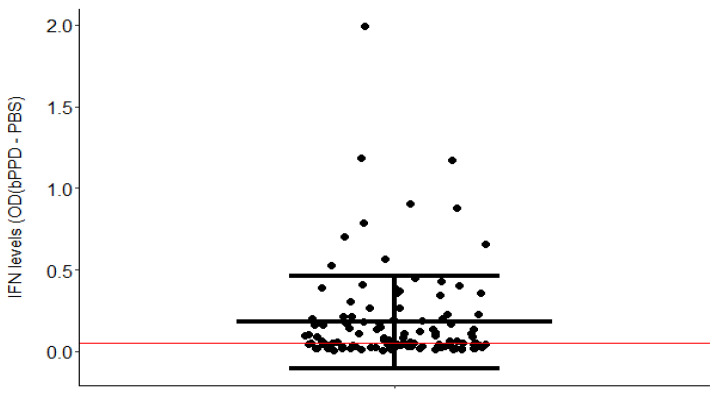
IFNγ production. Error plot of the levels of IFNɣ in stimulated blood samples from the 117 cows included in the study. The central line represents the mean of the group, and the whiskers represent the standard error. According to the interpretation criteria of the kit, 76 of the 117 cows included in the study were bTb-positive because the OD (bPPD-PBS) was >0.05 and the OD (bPPD-PBS) > OD (aPPD-PBS).

Ten animals had IFNɣ levels after blood stimulation with bPPD > 0.5. According to the interpretation criteria of the kit, 76 of the 117 cows included in the study had a positive IGRA result in response to bPPD. Figure 1 shows the mean of the IFNɣ production after blood stimulation with bPPD.

### 3.2. Heritability (h^2^) Estimate, Variance Components, and GWAS Results

**Figure 2:**
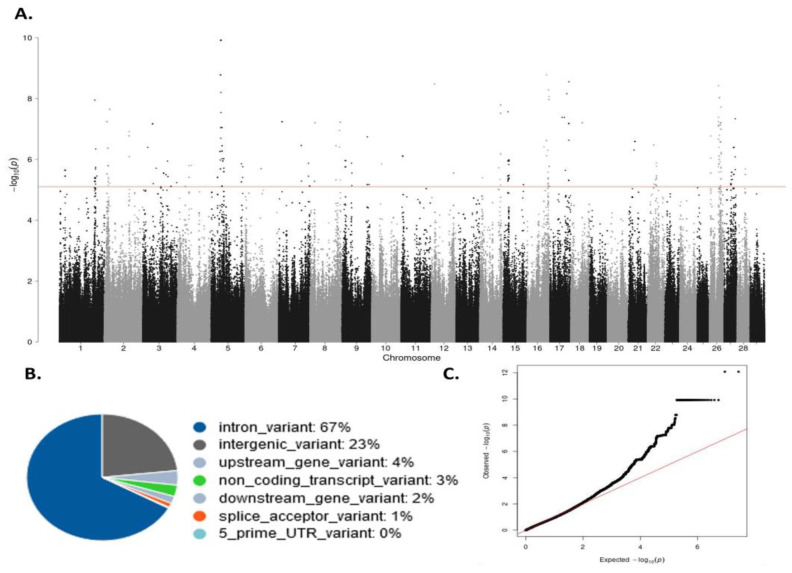
Results of the GWAS analysis. (**A**) Manhattan plot of the –log_10_ of the *p*-values of the association test between each SNP and the IFNɣ levels. Each dot represents one SNP. Chromosome localization of the SNPs is indicated on the x-axis. The horizontal red line is drawn at –log_10_ (5 × 10^−7^); (**B**) Genomic distribution of the 163 SNPs surpassing the threshold (*p*-value ≤ 5 × 10^−7^) according to the *Ensembl Variant Effect Predictor* (VEP); (**C**) Quantile–quantile plot comparing the observed distribution of –log (*p*-values) to the expected values under the null hypothesis.

The associations between genome-wide imputed SNPs and IFNɣ levels after blood stimulation with bPPD (OD (bPPD-PBS)) (N = 117) were analyzed using GCTA1.93.2. The heritability and variance components of the IFNɣ levels in the study population were estimated as 0.23 (σ_G_ = 0.01823, σ_e_ = 0.06146). The Manhattan plot shows –log_10_ (*p*-values) of the association test between IFNɣ levels and each SNP is represented in Figure 2A.

After clumping, the total number of SNPs that surpassed the significance criteria (P_FDR_ ≤ 0.05, *p*-value ≤ 5 × 10^−7^) was 163. The 163 significant SNPs were located on 20 different *Bos taurus* chromosomes. As seen in Figure 2B, most of the 163 SNPs were in intronic regions (67%), while the remaining identified SNPs were in intergenic regions (23%) or were upstream (4%) non-coding transcript variants (3%), or downstream (2%), variants. The quantile–quantile plot comparing the observed distribution of −log (*p*-values) to the expected *p*-values under the null hypothesis is shown in Figure 2C. The median inflation factor was 1.007588, which indicates the absence of population stratification.

### 3.3. SNPs, QTLs, and Candidate Genes Associated with IFNγ Production in Response to Bovine Tuberculin Stimulation

After clumping, a total of 163 SNPs, 72 QTLs, 197 candidate genes and 8 miRNAs (bta-mir-2285cf, bta-mir-2351, bta-mir-12005-2, bta-mir-12000, bta-mir-6121, bta-mir-6524, bta-mir-4680, and bta-mir-2399), were associated with production of IFNγ after stimulation with bPPD. *p*-values, QTLs positions, candidate genes, and miRNAs within each QTL are presented in Table 1. Pathway analysis failed to reveal significantly enriched biological processes and metabolic pathways when bovine-specific pathway data were considered.

The identified QTLs were distributed along the *Bos taurus* genome; chromosome 26 harbors the highest number of SNPs (N = 33). The QTL that harbored the SNP with the strongest association was on chromosome 26 (*p*-value = 8.36 × 10^−13^) and a b-value of 1.186. The b-values of all the identified SNPs were positive, which suggests that all the variants were associated with a significant IFNɣ production in response to *Mb*. 

**Figure 3:**
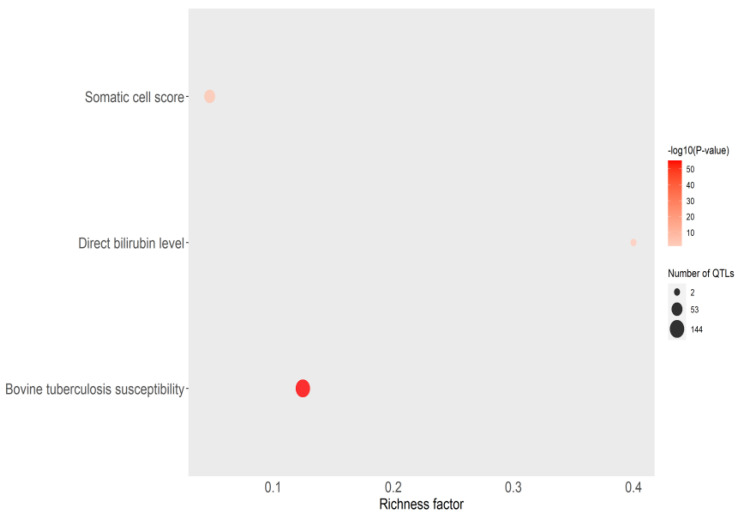
Bubble plot displaying the QTL enrichment results for health traits. The darker the red shade in the circles, the more significant the enrichment. The area of the circles is proportional to the number of QTLs. The x-axis shows a richness factor obtained by the ratio of the number of QTLs annotated and the total number of each QTL in the reference database.

Overlapping between the QTLs identified in the current study and QTLs previously associated with exterior, meat and carcass, production, reproduction, and health traits was observed. More specifically, overlapping between some of the identified QTLs and QTLs associated with 30 health traits was observed. The identified QTLs overlapped with 159 QTLs previously associated with bovine tuberculosis [29,49,50], 158 QTLs associated with somatic cell score [51,52,53,54,55,56,57,58,59], 105 QTLs associated with bovine respiratory disease susceptibility [60,61,62], and 14 QTLs associated with bovine paratuberculosis susceptibility [63,64,65,66,67,68]. QTLs enrichment analysis revealed the enrichment of 33 QTLs associated with exterior, meat and carcass, production, reproduction, and health (Supplementary Table S1). Of these, significant enrichment of QTLs previously associated with three health traits was observed, including bovine tuberculosis susceptibility (P-adjusted = 5.39 × 10^−56^), somatic cell score (P-adjusted = 4.43 × 10^−6^), and direct bilirubin level (P-adjusted = 0.04). The health traits identified in the QTL enrichment analysis are presented in Figure 3. 

We searched the animal genome database and discovered that 13% of the 91 identified candidate genes with a recognized gene name matched with candidate genes previously associated with bovine diseases such as bovine respiratory disease, mastitis, and bovine paratuberculosis. However, we did not find any matches with candidate genes associated with bovine tuberculosis. Some of the identified candidate genes, such as G Protein-Coupled Receptor Kinase 7 (*GRK7*), *ADAM metallopeptidase domain 9* (*ADAM9*), *furin*, *paired basic amino acid cleaving enzyme* (*FURIN*), and *Integrin Subunit Alpha V* (*ITGAV*) were previously associated with bovine respiratory disease susceptibility [62]. Both *FURIN* and *ITGAV* mediate *tumor growth factor β1* (*TGFβ1*) activation. Several candidate genes identified in our study were previously associated with somatic cell counts such as *Receptor tyrosine kinase-like orphan receptor* 1 (*ROR1*), *DAB adaptor protein 1* (*DAB1*), *PC-esterase domain containing 1B* (*PCED1B*), *Glucoside xylosyltransferase 1* (*GXYLT1*), *Solute carrier family 38* member 1 (*SLC38A1*), and *Toll-like receptor 4* (*TLR4*) [54,58].

Some of the identified candidate genes located on BTA11 (6,223,550–6,223,550 bp), such as *CCR4-NOT Transcription Complex*, *Subunit 11* (*CNOT11*), *RING-Type E3 Ubiquitin Transferase RNF149* (*RNF149*), and *Cellular Repressor Of E1A Stimulated Genes 2* (*CREG2*), were previously associated with the presence of PTB-associated multifocal lesions [69]. The *RFN149* negatively regulates *Mitogen-Activated Protein Kinase* (*MAPK*) cascade, and genetic variants in the vicinity of this gene were previously associated with the humoral immune response to MAP infection in dairy cattle [65]. *CNOT11* is linked to various cellular processes, including bulk mRNA degradation, miRNA-mediated repression, translational repression during translational initiation, and general transcription regulation. Candidate genes identified on BTA3 (63,633,887–63,633,887 bp) such as *Hyaluronan Binding Protein 2* (*HABP2*), *Nebulin Related Anchoring Protein* (*NRAP*), *Caspase 7* (*CASP7*), and *Pleckstrin Homology Domain Containing S1* (*PLEKHS1*) were previously associated with the presence of PTB-associated diffuse lesions [69]. HABP2 is involved in coagulation and fibrinolysis systems by activating coagulation factor VII and may function as a tumor suppressor, negatively regulating cell proliferation and cell migration. Mutations in this gene have been previously associated with nonmedullary thyroid cancer in humans [70] and susceptibility to venous thromboembolism due to thrombin defects [71]. CASP7 is a thiol protease involved in different programmed cell death processes, such as apoptosis, pyroptosis, or granzyme-mediated programmed cell death, by proteolytically cleaving target proteins and acts as a key regulator of the inflammatory response in response to bacterial infection by catalyzing the cleavage and activation of the sphingomyelin phosphodiesterase (SMPD1) in the extracellular milieu, thereby promoting membrane repair [72,73]. CASP7 also acts as an inhibitor of type I interferon production during pathogen-induced apoptosis by mediating cleavage of the antiviral proteins CGAS, IRF3, and MAVS, thereby preventing cytokine overproduction. Mutations in CASP7 might counteract these effects, resulting in cytokine overproduction and aberrant inflammation.

Some of the identified candidate genes matched with genes in the animal genome database previously associated with bovine paratuberculosis susceptibility, such as *Toll-like receptor 4* (*TLR4*) [66,67] and the *Periphilin 1* (*PPHLN1*), respectively. The PPHLN1 contributes to epidermal integrity and barrier formation and was previously associated with MAP resistance in Holstein cattle [63]. *Matrix Metallopeptidase 16* (*MMP16*) located on BTA14 was previously associated with positive ELISA, PCR, and bacteriological culture results for MAP infection detection [35], and it was also identified in the current study. MMP16 is a protein of the matrix metalloproteinase family involved in the breakdown of extracellular matrix components such as collagen type III and fibronectin in normal physiological processes. In the lung, several MMPs contribute to tissue homeostasis, such as MMP-7, -16, -19, -21, -24, -25, and -28 [74]. Allelic variants affecting MMPs might cause improper ECM remodeling and disease progression in pathological circumstances. 

Some of the identified candidate genes were previously associated with several bovine traits, highlighting their importance not just in health but also in milk production, fertility, body conformation, and length of productive life. For instance, *TLR4* was identified in our study and was previously found to be associated with 18 bovine traits, some of them health traits including basophil and lymphocyte number, somatic cell counts, and MAP infection susceptibility [66,67]. *TLR4* plays a fundamental role in pathogen recognition and activation of innate immunity, and mutations in this receptor might result in inadequate antigen recognition and processing, leading to immune tolerance. 

By searching the human GWAs catalog, we found that *PC-esterase domain containing 1BP* (*PCED1B*), a hydrolase involved in macrophage apoptosis and autophagy, and *ArfGAP With SH3 Domain*, *Ankyrin Repeat And PH Domain 1* (*ASAP1*) were the only candidate genes identified in our study that were previously associated with human tuberculosis [75]. *PCED1B* was also previously associated with bovine respiratory disease susceptibility [62]. Susceptibility to human tuberculosis was associated with variants in the *ASAP1* gene encoding a regulator of dendritic cell migration [76]. Most of the 91 candidate genes with a recognized gene symbol were included in the InnateDB database and had a role in signaling pathways involved in the bovine immune response against microbial infections, including *TLR4* and *programmed cell death protein 4* (*PDCD4*), among others. 

### 3.4. Protein-to-Protein Interaction Analysis

**Figure 4:**
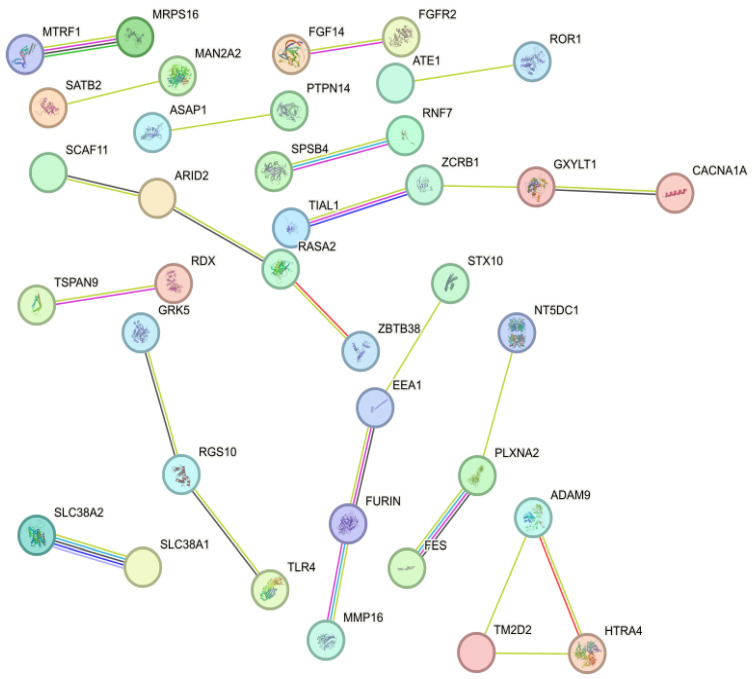
Protein-to-protein network analysis using the identified candidate genes. Individual nodes represent proteins with relationships represented by edges. The green lines represent gene neighborhood, red lines represent gene fusions, blue lines represent gene co-occurrence, black lines represent co-expression, pale blue lines represent homology, and purple lines represent experimentally determined interaction. The candidate proteins with no associations to other proteins were hidden.

The 197 identified candidates were analyzed for protein-to-protein interactions. The analysis revealed five networks: GRK5-RGS10-TLR4, STX10-EEA1-FURIN-MMP16, TIAL1-ZCRB1-GXYLT1-CACNA1A, TM2D2-ADAM9-HTRA4, and SCAF11-ARID2-RASA2-ZBTB38 (Figure 4).

### 3.5. gEBVs for IFNγ Production and Correlations with Other Bovine Traits

gEBVs for IFNγ production were calculated for each animal in the study population and then predicted in a larger population of 1739 Friesian cattle. Correlations between the gEBVs for IFNγ levels in this larger population and 65 phenotypes and traits included in the evaluations of Spanish Holstein cattle were calculated with Spearman’s correlation test. For the phenotypes and traits with a significant correlation (*p* ≤ 0.05) with IFNγ production, the absolute value of Spearman’s correlation index was lower than 0.12.

## 4. Discussion

The use of cellular immunity traits in genetic linkage studies in Holstein cattle is scarce. Recently, a strong effect of host genetics on IFNγ production in response to the avian tuberculin was observed [33]. In the current study, we measured the IFNγ production in response to the bPPD in 343 Holstein cows in two steps consisting of (i) incubating whole blood samples from the selected animals with bPPD and (ii) detecting the presence of IFNγ released by sensitized lymphocytes in the whole blood sample to indicate a CMI to the specific antigen. The number of samples (N = 117) used in this study could be considered small in comparison with traditional GWAS analysis. The main limitation that arises from having a small sample size is the decrease in statistical power. However, the assessment of IFNɣ levels in stimulated blood is a functional and controlled trait that allowed us to identify 163 SNPs significantly associated with high IFNɣ production. Similar reductionist phenotypes, such as the assessment of the macrophages’ performance by measuring MAP load within MDMs, used only 61 samples [77]. 

Previous studies quantified the genetic variation for bTb in different cattle populations and countries using a variety of trait definitions and reported heritability estimates that ranged between 0.06 and 0.18 [22,23,24,25]. In the current study, we demonstrated that IFNγ production in response to *Mb* is, at least to some extent, dependent on host genetics (h^2^ = 0.23). Putative QTLs associated with *Mb* infection in Holstein cattle have been reported on the *Bos taurus* chromosome 1 (BTA1) [28], BTA2 and BTA13 [22], BTA6 [78,79], BTA22 [80], and BTA23 [28,49]. In most of these studies, case cows had a positive CITT, histopathological lesions, and a positive bacteriological culture result, and all other cattle present in the herd were considered control cows. In the current study, none of the identified genomic regions associated with IFNγ production were located on BTA23. Differences between studies may be due to differences in the phenotypes used, population structure, methodologies used across studies, and the large polygenic inheritance of the trait. In addition to the individual QTL differences between studies, our QTLs enrichment analysis revealed a total of three health traits with QTLs overlapping the QTLs identified in our study, including bovine tuberculosis susceptibility as the most significant trait. This finding suggests that the capacity to produce a strong IFNγ in response to *Mb* is an indicator of bTb susceptibility in cattle. Previous studies demonstrated that strong IFNγ production correlated with bTb pathology in cattle and badgers [14].

We identified 163 SNPs, 72 QTLs, 197 candidate genes, and 8 miRNAs associated with high IFNγ production in response to *Mb* infection. The 163 significant SNPs were located on 20 different *Bos taurus* chromosomes. This suggests that IFNɣ production is a polygenic trait depending on many SNPs located on different chromosomes. The identified QTLs overlapped with a total of 159 QTLs in chromosomes 3, 5, 16, 17, 22, and 27 that were previously associated with bTb susceptibility [29,49,50]. Our study identified candidate genes that might result in an inadequate recognition of *Mb* antigens, reduced autophagy, inflammasome activation, uncontrolled extracellular matrix degradation, and reduced immune cell migration. The *RAS p21 protein activator 2* (*RASA2*) gene identified in our study encodes a protein that has been previously demonstrated to serve as a tumor suppressor in melanoma, and therefore, mutations affecting *RASA2* might be associated with uncontrolled inflammation [81]. In the protein network analysis, we found a network (*SCAF11-ARID2-RASA2-ZBTB38*) centered in RASA2, together with the chromatin remodeling factor *ARID2*, also mutated in various cancer types [82].

Another candidate gene identified in our study was the TLR4 receptor. In the protein network analysis, we found a network (*GRK5-RGS10-TLR4*) containing the *TLR4* gene. G Protein-Coupled Receptor Kinase 5 (GRK5) regulates the motility of polymorphonuclear leukocytes, and TLR4 recognizes pathogen-associated molecular patterns (PAMPs) that are expressed in mycobacteria and mediate the production of cytokines necessary for the development of effective immunity. Polymorphisms in the *TLR4* gene have been shown to affect MAP recognition and have been associated with increased susceptibility of cattle to paratuberculosis [83]. A recent study has shown that knocking out the *TLR4* gene in bovine MAC-T cells enhances inflammation in response to MAP infection [84]. TLR4 mutant C3H/HeJ mice can control *Mb* BCG infection as well as C3H/HeOUJ control mice, with efficient macrophage recruitment and activation, but they have arrested body weight and develop chronic exacerbated inflammation at later stages of infection [85]. Under a similar bacterial burden, inflammation was exacerbated and persisted longer in TLR4-deficient mice, suggesting that a “switch off” signal for inflammation was missing in the absence of a functional TLR4. Our study is the first to identify *RASA2*, *GRK5*, and *TLR4* as candidate genes associated with strong IFNγ production in response to *Mb* infection. Mutations affecting these genes might cause improper Mb antigen recognition and uncontrolled inflammation. 

Interestingly, the PCED1B, a hydrolase involved in apoptosis and autophagy of infected macrophages, was identified in our study, and it was also associated with human Tb susceptibility [75]. PCED1B plays a crucial role in apoptosis and autophagy, which are important mechanisms of innate immunity against *Mtb* [86]. Mutations in *PCED1B* might result in impaired autophagy and increased inflammasome activation in both human and bovine Tb. The *ASAP1* gene was identified in our study and previously associated with human Tb [76]. Susceptibility to human tuberculosis is associated with variants in the *ASAP1* gene, which encodes a regulator of dendritic cell (DC) migration. SNPs in *ASAP1* were significantly associated with Tb in the Russian population [78], the Han Chinese population [87], and the Xinjiang Muslim population [88]. Impaired migration of mycobacteria-infected DCs, caused by the genetically determined excessive reduction of ASAP1 expression, may contribute to human and bovine tuberculosis. This may be one of the mechanisms that lead to the slow migration of DCs to lymph nodes and the delay of the adaptive immune response during the early stages of tuberculosis infection [76].

A total of eight miRNAs (bta-mir-2285cf, bta-mir-2351, bta-mir-12005-2, bta-mir-12000, bta-mir-6121, bta-mir-6524, bta-mir-4680, and bta-mir-2399) were associated with high production of IFNγ after stimulation with bPPD. MiRNAs are important regulators of innate and adaptative immune responses. More specifically, several miRNAs have been found to regulate T-cell functions or the innate function of macrophages, dendritic cells, and NK cells [89,90]. For example, bta-miR-4680 is expressed in bovine alveolar macrophages [91], suggesting its potential implication in infectious respiratory diseases. On the other hand, the identified bta-miR-2285cf is part of the bta-miR-2285 family, with 40 members spanning the entire bovine genome, which is expressed in response to Gram-positive bacteria infection [92] and in macrophages infected with *Streptococcus agalactiae* [93]. bta-mir-2399 is part of the repertoire of bovine miRNAs and miRNAs-like small regulatory RNAs expressed upon viral infections [94]. Future functional studies are required to confirm the association of these miRNAs, IFNγ production, and susceptibility to *Mb* infection. 

Despite the vast research about the immune response mechanisms of human tuberculosis caused by *Mtb*, the knowledge of bovine tuberculosis’s immunology, particularly regarding the innate immune response, remains scarce. Our study advances the understanding of the role of bovine IFNγ in mycobacterial infections. It was interesting to find that none of the SNPs and QTLs identified in the current study overlapped with QTLs associated with strong production of IFNγ in blood samples stimulated with aPPD [33]. These findings show that different genetic variations are associated with enhanced IFNγ production in response to *MAP* or *Mb* [95]. Early after MAP infection, a strong IFNγ production correlates with resistance [33]. In contrast, strong IFNγ production in response to bovine tuberculin correlates with bTb susceptibility in more advanced stages of *Mb* infection. An IGRA to distinguish between *Mb* infection and bTb disease has not yet been developed [96].

Our results open the possibility of ranking Holstein cows based on predicted IFNɣ production, which would allow producers to select cattle less susceptible to bTb, ultimately reducing the prevalence of the disease, preventing economic losses, and increasing the length of cattle’s productive life. Genetic selection of less susceptible cattle would be particularly useful in low and middle-income countries where test-and-slaughter-based control programs are unfeasible. Importantly, the correlations between IFNɣ production and other animal traits were close to zero, suggesting that selective breeding to reduce animal susceptibility to bTb would not compromise improvements in other traits [25]. 

## 5. Conclusions

We report a new phenotype, the production of IFNɣ in response to bovine tuberculin, for evaluating susceptibility to bTb in dairy cows. The identified SNPs, QTLs, and candidate genes revealed an association between high IFNɣ levels after stimulation of blood samples with bPPD and host genetic susceptibility to bTb. QTLs enrichment analysis revealed a total of three health traits, with QTLs overlapping the QTL identified in this study, including bovine tuberculosis susceptibility, which is the most significant trait. To our knowledge, this is the first study revealing a genetic association between bovine IFNγ production and candidate genes involved in various crucial biological processes, including the recognition of bacterial antigens, apoptosis, autophagy, extracellular matrix remodeling, and the migration of immune cells. Furthermore, our results have important implications regarding the use of genetic evaluations for IFNγ production complementing bTb control.

## Figures and Tables

**Table 1:** QTLs, candidate genes, and miRNAs associated with high levels of IFNɣ in response to bovine tuberculin.

BTA ^1^	QTL Start (bp)	QTL End (bp)	Peak *p*-Value	Genes in QTL ^2^	No. SNPs in QTL
1	124,889,999	124,889,999	4.05 × 10^−6^	*ENSBTAG00000059771, DIPK2A, ENSBTAG00000064298, ENSBTAG00000060304, SLC9A9*	1
1	125,545,373	125,555,920	5.26 × 10^−6^		2
1	127,196,414	127,784,694	2.06 × 10^−6^	*GRK7, RNF7, RASA2, U6, bta-mir-2285cf, ZBTB38, PXYLP1, ENSBTAG00000050470, ENSBTAG00000059581, SPSB4*	5
1	20,524,991	20,585,846	2.29 × 10^−6^		4
2	13,171,064	13,171,064	1.57 × 10^−6^		1
2	14,086,412	14,102,103	1.64 × 10^−6^	*ENSBTAG00000053080, ENSBTAG00000058959, PDE1A*	2
2	15,276,423	15,276,423	3.92 × 10^−6^	*ENSBTAG00000064762*	1
2	19,826,914	19,958,921	2.24 × 10^−8^	*ENSBTAG00000069400, ENSBTAG00000069332, ENSBTAG00000064902*	2
2	87,618,721	87,987,549	1.21 × 10^−7^	*SATB2*	2
2	9,485,693	9,772,662	5.72 × 10^−8^	*FAM171B, ZSWIM2, ITGAV, bta-mir-2351*	5
3	47,301,950	47,301,950	1.94 × 10^−6^		1
3	63,633,887	63,633,887	7.87 × 10^−6^		1
3	73,139,474	73,139,474	2.82 × 10^−6^	*NEGR1*	1
3	81,693,793	81,693,793	3.16 × 10^−6^	*ROR1*	1
3	87,101,478	87,101,478	1.14 × 10^−6^	*ENSBTAG00000066077*	1
3	88,629,927	88,629,927	3.65 × 10^−6^	*DAB1*	1
4	28,439,718	28,439,718	7.73 × 10^−6^	*POLR1F, TMEM196*	1
5	106,699,607	106,710,516	5.50 × 10^−6^	*TSPAN9, ENSBTAG00000060879*	2
5	22,620,852	22,963,465	3.86 × 10^−6^	*PLEKHG7, U6, EEA1, ENSBTAG00000065664, ENSBTAG00000068135*	5
5	31,376,781	31,376,781	5.49 × 10^−7^	*OR8S27, OR8S25, OR8S10*	1
5	32,917,758	33,096,745	1.66 × 10^−9^	*ENSBTAG00000059572, ENSBTAG00000038027, ENSBTAG00000060945, ENSBTAG00000067834, PCED1B*	2
5	33,790,732	34,674,103	1.21 × 10^−10^	*ENSBTAG00000062330, ENSBTAG00000062600, ENSBTAG00000058122, SLC38A2, ENSBTAG00000067121, SLC38A1, SCAF11, ARID2, ENSBTAG00000058264, ENSBTAG00000069144, ENSBTAG00000067082*	6
5	37,435,087	38,572,334	8.97 × 10^−8^	*ENSBTAG00000067640, SNORA62, bta-mir-12005-2, PRICKLE1, ENSBTAG00000060964, PPHLN1, ZCRB1, YAF2, GXYLT1*	8
5	44,552,213	44,780,908	9.47 × 10^−7^	*U6, LYZ, CPSF6, ENSBTAG00000066263, ENSBTAG00000069891, ENSBTAG00000057583, ENSBTAG00000064106, ENSBTAG00000002741, ENSBTAG00000044636, ENSBTAG00000064663, ENSBTAG00000069326*	3
6	56,297,100	56,297,100	2.00 × 10^−6^		1
7	109,402,049	109,402,049	7.49 × 10^−6^		1
7	12,351,770	12,389,993	5.77 × 10^−8^	*CACNA1A, bta-mir-12000, ENSBTAG00000060372, ENSBTAG00000062006, IER2, STX10*	2
7	80,281,173	80,281,173	3.50 × 10^−7^	*TENM2*	1
8	106,878,958	107,205,388	5.95 × 10^−8^	*TLR4, ENSBTAG00000068729, ENSBTAG00000061668*	3
8	16,064,815	16,064,815	5.10 × 10^−6^	*LINGO2, ENSBTAG00000066439*	1
8	16,591,629	16,755,960	5.32 × 10^−6^	*ENSBTAG00000067505, ENSBTAG00000060335, IFNK*	3
8	92,008,532	92,008,532	4.67 × 10^−6^		1
9	11,599,785	11,938,116	1.09 × 10^−6^	*RIMS1*	2
9	33,772,705	33,772,705	1.33 × 10^−6^	*RFX6*	1
9	34,608,918	34,906,969	2.80 × 10^−6^	*NT5DC1, SYNE1*	2
9	89,314,702	89,389,719	6.70 × 10^−6^	*SYNE1, ENSBTAG00000052173*	2
9	95,898,153	95,898,153	6.69 × 10^−6^	*ENSBTAG00000052316*	1
11	6,223,550	6,223,550	7.77 × 10^−7^	*CNOT11, ENSBTAG00000067224, SNORD89, ENSBTAG00000064562, RNF149, CREG2*	1
12	11,410,693	11,410,693	3.30 × 10^−9^	*KBTBD7, MTRF1*	1
12	78,341,192	78,341,192	2.79 × 10^−6^	*FGF14*	1
14	10,240,609	10,240,609	4.03 × 10^−6^	*ASAP1*	1
14	74,566,478	74,566,478	1.61 × 10^−8^	*MMP16*	1
15	19,232,962	19,232,962	4.12 × 10^−6^		1
15	20,351,354	20,406,919	1.04 × 10^−6^	*RDX, ENSBTAG00000068159, ENSBTAG00000066977*	2
15	72,080,092	72,080,539	6.78 × 10^−6^		2
16	59,681,912	59,681,912	3.82 × 10^−7^		1
16	68,538,475	68,542,979	2.40 × 10^−7^	*KCNK2*	2
16	69,071,657	69,071,657	1.66 × 10^−9^	*PTPN14*	1
16	73,402,526	73,402,526	5.00 × 10^−7^	*SYT14, ENSBTAG00000065259, ENSBTAG00000064536, UTP25*	1
16	74,037,392	74,222,831	2.63 × 10^−6^		3
16	74,743,716	75,220,658	5.16 × 10^−9^	*ENSBTAG00000042659, PLXNA2, bta-mir-6121*	5
16	77,902,129	77,902,129	1.70 × 10^−6^	*ENSBTAG00000059177*	1
17	50,628,634	50,628,634	4.15 × 10^−8^	*TMEM132B*	1
17	59,585,737	59,585,737	6.92 × 10^−9^	*U2*	1
17	66,464,708	66,600,043	2.81 × 10^−9^		5
21	21,803,104	21,803,104	4.91 × 10^−7^	*MAN2A2, FES, FURIN, ENSBTAG00000057133, BLM*	1
21	25,085,953	25,085,953	2.56 × 10^−7^	*BTBD1, ENSBTAG00000066584, ENSBTAG00000063590*	1
22	27,941,466	28,433,940	4.60 × 10^−6^	*ENSBTAG00000069336, ENSBTAG00000069185, PDZRN3*	3
22	31,251,423	31,507,701	1.30 × 10^−6^	*ENSBTAG00000064803, ENSBTAG00000061892, MDFIC2*	4
26	31,413,153	31,413,153	5.68 × 10^−8^	*RBM20, ENSBTAG00000061228, ENSBTAG00000061228, ENSBTAG00000059480, PDCD4, bta-mir-6524, bta-mir-4680*	1
26	31,943,002	32,482,052	8.36 × 10^−13^	*ENSBTAG00000059653, ENSBTAG00000062595, ENSBTAG00000066814, ENSBTAG00000064546, ENSBTAG00000059196, ENSBTAG00000061789*	8
26	33,800,182	34,212,369	7.72 × 10^−7^	*HABP2, NRAP, CASP7, PLEKHS1*	4
26	35,012,876	35,056,491	9.30 × 10^−9^	*ABLIM1*	3
26	37,967,031	38,185,671	6.19 × 10^−8^		5
26	39,549,032	39,664,208	6.19 × 10^−8^	*ENSBTAG00000063944, ENSBTAG00000056024, RGS10, TIAL1*	3
26	40,347,155	41,671,467	1.90 × 10^−8^		9
27	23,351,726	23,817,855	6.29 × 10^−6^	*ENSBTAG00000060821, ENSBTAG00000068762, C27H8orf48, DLC1*	2
27	25,170,223	25,252,376	3.51 × 10^−6^	*ENSBTAG00000060632, PPP1R3B, U6*	2
27	26,556,329	26,560,206	2.63 × 10^−6^	*RBPMS, ENSBTAG00000053684, bta-mir-2399*	2
27	29,107,302	29,107,302	4.53 × 10^−6^	*ENSBTAG00000057376, FUT10, ENSBTAG00000054272*	1
27	34,187,524	34,187,953	4.04 × 10^−7^	*HTRA4, TM2D2, ENSBTAG00000068068, ADAM9*	2
28	29,347,932	29,347,932	2.05 × 10^−6^	*MRPS16, CFAP70, ANXA7, ENSBTAG00000054455*	1

^1^ Chromosome QTL location, ^2^ Candidate genes within the identified QTL.

## Data Availability

The original contributions presented in this study are included in the article/Supplementary material. Further inquiries can be directed to the corresponding author. Sequence data used in this study for the imputation to WGS are owned by the 1000 Bull Genomes Project Consortium. Individual genotype data used in this study are managed by a third party, the Spanish Friesian Cattle National Federation (CONAFE). Request for individual genotype data can be made to CONAFE, Ctra. De Andalucia, km. 23,600–28,340 Valdemoro, Madrid, Spain; email: conafe@conafe.com; phone: +34-(91)-8952412; Fax: 918-951-471; website: www.conafe.com. CONAFE is a member of the Eurogenomics Cooperative U.A.

## Supplementary Materials

The following supporting information can be downloaded at: https://www.mdpi.com/article/10.3390/ijms25116165/s1.
